# The Current Role of Lymph Node Dissection in Nonmetastatic Localized Renal Cell Carcinoma

**DOI:** 10.3390/jcm12113732

**Published:** 2023-05-29

**Authors:** Megan Ngai, Thenappan Chandrasekar, Gennady Bratslavsky, Hanan Goldberg

**Affiliations:** 1 Urology Department, SUNY Upstate Medical University, Syracuse, NY 13210, USA; 2 Department of Urology, University of California, Orange, CA 92868, USA

**Keywords:** renal cell carcinoma, kidney cancer, lymph node dissection

## Abstract

Purpose: To explore the current role of lymph node dissection (LND) in the management of nonmetastatic localized renal cell carcinoma (RCC). Background: There is currently no proven benefit of LND in the setting of RCC, and its role remains controversial because of conflicting evidence. Patients who may benefit from LND are those at greatest risk of nodal disease, but the tools used to predict nodal involvement are limited due to unpredictable retroperitoneal lymphatics. The indications, templates, and extent of LND are also not standardized, adding to the ambiguity of current guidelines surrounding its use. Evidence Acquisition: A PubMed search of the literature from January 2017 to December 2022 was conducted using the search terms “renal cell carcinoma” or “renal cancer” in combination with “lymph node dissection” or “lymphadenectomy”. Case studies and editorials were excluded, whereas studies investigating the therapeutic effect of LND were classified as either demonstrating a benefit or no benefit. References of the studies and review articles were also searched for notable studies and findings that were outside the five-year literature search. The studies in this review were restricted to the English language. Results: Only a number of studies in recent years have found an association between the extent of LND and increased survival. Most studies do not indicate an associated benefit, and some even suggest a negative effect on survival. Most of these studies are retrospective. Conclusion: The therapeutic value of LND in RCC is still unclear, and although prospective data are needed, its declining rates and emerging new therapies make this unlikely. A better understanding of renal lymphatics and improved detection of nodal disease may help determine the role of LND in nonmetastatic localized RCC.

## 1. Introduction

It is widely accepted that lymph node involvement (LNI) in the setting of nonmetastatic renal cell carcinoma (RCC) is generally associated with poor survival outcomes. Recent studies have found that patients with the node-positive disease show comparable survival outcomes to those with metastatic RCC [1,2]. This makes lymph node dissection (LND) a critical procedure for prognostic and staging purposes in the management of RCC, but its therapeutic benefit remains controversial.

First, initial support for the oncologic role of LND reported by Robson et al. [3] has largely been challenged by conflicting data, and the European Organization for Research and Treatment of Cancer (EORTC) trial number 30881 remains the only prospective randomized, controlled trial to evaluate the benefit of LND in RCC [4]. This randomized trial assessed whether there was a survival benefit among patients who underwent nephrectomy with LND in comparison with those who underwent nephrectomy alone. The results showed no oncologic advantage of LND, but only 4% of the cohort had a nodal disease, and a majority had low-stage tumors. Other retrospective studies with similar findings suggest that these results may only apply to low-risk cases or those without the nodal disease [5,6,7]. For now, patients with clinically negative lymph nodes or low-stage tumors (T1–T2) are not recommended to undergo LND because it offers limited staging information and no survival benefit [4].

It is still debated whether LND has a possible therapeutic advantage for patients with nonmetastatic RCC and LNI. Several studies have attempted to clarify its role and found improved survival outcomes for certain RCC patients who underwent LND, but other studies showed no difference, regardless of high-risk disease [8,9,10]. Nonetheless, most studies by both proponents and opponents of LND in nonmetastatic patients have been retrospective in nature and based on heterogeneous data due to inconsistent LND templates and indications, warranting further prospective studies.

The lack of proven benefits and controversy surrounding LND are reflected in current guidelines and summarized in Table 1. The American Urological Association (AUA) recommends LND of all clinically positive nodes only for staging purposes [11]. Similarly, the guidelines from the European Association of Urology (EAU) suggest LND during nephrectomy when there are clinically enlarged lymph nodes for staging and prognostic reasons and do not recommend it in the case of organ-confined disease [12]. The National Comprehensive Cancer Network (NCCN) recommends LND in the case of suspected lymphadenopathy on preoperative imaging and palpable or enlarged lymph nodes [13]. These guidelines recommend LND only in patients with nodal disease and underscore its staging and prognostic value. However, they do not mention any oncologic benefit nor indicate which patients may benefit. Furthermore, current data show that the number of patients with RCC and suspected lymphadenopathy does not match the rates of LND being performed, suggesting possible overutilization of LND for patients with the low-stage disease and/or underutilization for patients with the high-risk disease [9,14]. Given this discrepancy, it is not only difficult to elucidate the possible therapeutic benefit of LND but also challenging to identify high-risk patients who may benefit from it, especially if patients with suspected lymphadenopathy are not undergoing LND as clinically recommended. This is further complicated by the fact that clinical lymph node status does not always correlate with pathological lymph node status, where about 70% of clinically suspicious lymph nodes do not show lymph node invasion upon final pathological report [15].

Additionally, the American Joint Committee on Cancer (AJCC) currently has two definitions of stage III disease, those with lymph-node positive (pT1-3N1M0) and lymph node-negative (pT3NX-0M0) disease [16]. This essentially groups patients with T3 disease with and without LNI in the same category, despite a significantly worse prognosis for those with nodal disease [1,2,17]. There is increasing support for the reclassification of pT3 RCC with nodal disease as stage IV disease. Further stratification in the TNM staging system may better identify high-risk candidates for LND and determine its potential benefit.

Third, the complex renal lymphatic drainage into the retroperitoneal lymph nodes remains one of the biggest issues in the standardization of LND templates and results in an alarming heterogeneity of performed LND templates across various studies. Unlike most cancers that disseminate via locoregional lymphatics, RCC exhibits both early hematogenous dissemination and lymphomatous dissemination, which deviate from proposed locoregional lymphatic drainage patterns [5,18]. The unpredictability of retroperitoneal lymphatics from the kidney poses a significant barrier in determining the possible oncologic benefit of LND. Common LND templates for right- and left-sided RCC may not sufficiently capture all sites of lymphatic drainage for patients with the high-risk disease [19]. This raises the question of whether the current extent of LND is even sufficient to confer a therapeutic benefit in patients with nodal disease and calls for standardization of LND templates. However, determining its potential benefit will only prove more challenging with the declining rates of LND [20]. Its eventual role in the management of nonmetastatic RCC will surely change with novel adjuvant and neoadjuvant immunotherapies and other emerging therapies.

Regardless, it is clear LND is beneficial for staging purposes and is therefore prognostic as well. Whether it offers a survival advantage for RCC patients with and without nodal disease remains unclear and necessitates further studies. This review will explore the existing literature regarding the controversial therapeutic use of LND in the management of nonmetastatic RCC and highlight current challenges.

## 2. Evidence Acquisition

A PubMed search of the literature published within the last five years (from January 2017 to December 2022) was performed using keywords including “renal cell carcinoma” or “renal cancer” in combination with “lymph node dissection” or “lymphadenectomy”. The studies were selected for this review if they included patients with nonmetastatic RCC or LNI. The studies were then classified as those that supported the therapeutic benefit of LND and those that were against LND. References of these studies and review articles were also hand searched for other relevant studies to include significant studies that were not within the five-year search range. Case studies, editorials, and articles not in English were excluded. The benefit of LND was assessed with survival outcomes, such as progression-free survival, cancer-specific survival (CSS), overall survival (OS), cancer-specific mortality (CSM), and all-cause mortality (ACM), as outlined in Table 2.

### 2.1. Relevant Anatomy and Lymph Node Dissection Templates

The unpredictable lymphatic drainage pattern from the kidney and heterogeneity of the LND templates performed across various institutions pose a significant challenge in determining the therapeutic value of LND and establishing a standard template. Current templates are based on primary lymphatic drainage, first described in the early 1900s [34]. Lymphatics from the right kidney drain anteriorly into the paracaval, precaval, retrocaval, and interaortocaval nodes and posteriorly into the paracaval, retrocaval, and interaortocaval nodes; lymphatics from the left kidney drain anteriorly into the paraaortic and preaortic nodes and posteriorly into the paraaortic and retroaortic nodes [35,36]. These primary landing sites serve as the basis for the most common LND templates [19] but are not representative of all renal lymphatics since in vivo drainage studies have shown high individual variability [18]. Kuusk et al. found sentinel nodes that were beyond these locations in 35% of patients, including supradiaphragmatic landing sites.

Other mapping studies have found direct drainage into the thoracic duct [37,38] and possible drainage at the level of renal veins (when assessed in rodents and primates), which may explain the hematogenous spread of RCC [34]. This was reinforced by a retrospective study from Crispen et al. who defined an LND template based on locations of lymph node metastases (LNM) for patients with two or more high-risk features for nodal disease. Patients were considered high-risk if an intraoperative assessment of the primary tumor revealed a score of nuclear grade 3 or 4, with sarcomatoid features, size ≥ 10 cm, tumor stage pT3 or pT4, or coagulative tumor necrosis. They found that the locations of LNM were mostly consistent with the proposed primary landing sites but also noted that nodal disease on the ipsilateral hilar lymph node was negative 45% of the time [39]. This suggests that disease staging may be inaccurate or under-staged if nodal sampling is limited to the hilar lymph nodes. Consequently, their recommended LND template included the paracaval and interaortocaval lymph nodes for patients with right-sided tumors and paraaortic and interaortocaval lymph nodes for patients with left-sided tumors from the crus of the diaphragm to the common iliac artery. Altogether, their findings have major implications for LND in the management of RCC for both staging and possible therapeutic purposes.

**Figure 1:**
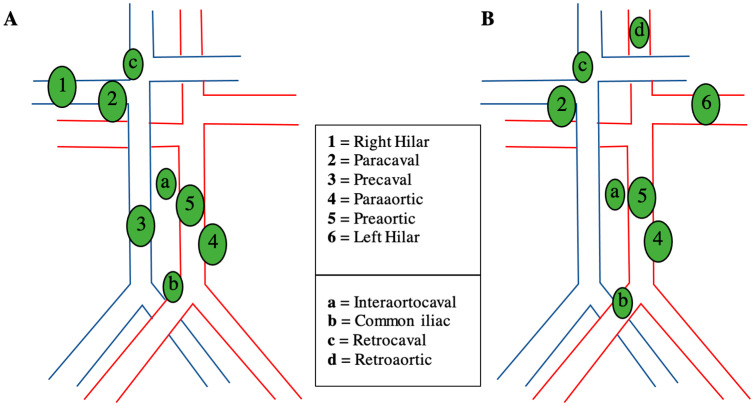
The most common templates for lymph node dissection (LND) in renal cell carcinoma (RCC) as described by Campi et al. [19] from the crus of the diaphragm to the aortic bifurcation. (**A**) For right-sided tumors, this includes the hilar, paracaval, and precaval lymph nodes, with a few studies extending to the retrocaval, pre/paraaortic, common iliac artery, and interaortocaval lymph nodes. (**B**) For left-sided tumors, this includes the hilar, paraaortic, and preaortic lymph nodes, with a few studies extending to the retroaortic, paraaortic, common iliac artery, and interaortocaval lymph nodes.

Campi et al. conducted a systematic review of the literature to find the most common LND templates, and these are represented in Figure 1. For right-sided tumors, the most dissected templates included the hilar, paracaval, and precaval lymph nodes from the crus of the diaphragm to the aortic bifurcation. For left-sided tumors, they included the hilar, preaortic, and paraaortic lymph nodes with the same boundaries. Only a few studies extended right-sided dissections to include the interaortocaval, retrocaval, common iliac, or pre/paraaortic lymph nodes and the interaortocaval, retroaortic, common iliac, or paracaval lymph nodes for left-sided dissections [19]. Still, their study was limited by the lack of consistency regarding the indications and extent of LND. Information was either not reported or not standardized in the 25 studies they analyzed. This reiterates the ambiguity of LND and the claim that the most used templates may not adequately capture the extent of nodal disease. The complexities of renal lymphatics are still not understood, and it was only recently revealed that the side or location of LNI does not contribute to increased risk of nodal disease at surgery or nodal progression at follow-up [40]. Additional in vivo mapping studies are needed to illuminate the role of LND and perhaps develop a universal template.

### 2.2. Evidence Supporting Lymph Node Dissection

Improved survival was found among a small subset of patients who underwent LND at the time of nephrectomy. Gershman et al. found durable survival for patients with isolated LNI, and similar results were shown by Tachibana et al. for patients with limited nodal disease (1–2 positive lymph nodes) who underwent LND [21,22]. Their findings suggest that patients with less aggressive RCC may benefit from LND, but only a few studies have been able to establish a link between LND and possible survival advantages. Gershman et al. noted that no standardized LND template was used, only the total number of nodes removed and positive nodes removed were reported [21]. Without knowing the specific nodes removed, the applications and findings of their study are limited since it is unclear whether the LND sufficiently captured the disease. Similarly, Tachibana et al. did not define their limited LND template, but they defined a left-sided LND template to include the paraaortic nodes and a right-sided LND template to include the paracaval and interaortocaval nodes [22]. Even though their template LND includes the primary landing sites, their results have limited application for patients with more than two positive nodes. Moreover, their findings are limited by their small sample size of 138 patients and 45 patients, respectively.

Several retrospective studies have found an association between improved survival and the extent of LND. In 2011, Whitson et al. conducted a population-based study and found significantly improved survival with an increasing number of lymph nodes removed, alluding to a possible benefit of LND in patients with node-positive RCC [23]. They postulated that LND might contribute to survival through the removal of micro-metastatic disease. Despite being one of the few studies with a larger sample size (*n* = 9586) that support LND, this study was criticized for heavily relying on imputation to derive the missing tumor grade for 28% of their cohort. Though their findings demonstrate quantitative data, where an increasing number of lymph nodes removed show improved survival, the study lacks qualitative data that specifically define the positive nodes for a possible template. When Sun et al. reanalyzed their data and excluded the missing data, increasing the extent of LND no longer endowed a protective effect on CSM for patients with nodal disease [41].

Even so, a later study by Capitanio et al. also showed improved CSS with an increasing number of lymph nodes removed for patients with pT4 RCC [24]. Specifically, the risk of mortality decreased by 8% for every lymph node removed. Subsequent studies by the same authors also demonstrated improved CSS and metastatic progression-free survival (MPFS) with LND in patients with pT2-4 stage, bulky tumors (>10 cm), or sarcomatoid components [25]. However, their findings are once again limited by an especially small sample size of 44 patients and possibility of missing additional nodal disease. Their extended LND template for left-sided tumors included the paraaortic and preaortic nodes and for right-sided tumors included the retro/precaval nodes [24]. Given that 35% of patients have sentinel nodes beyond these sites [18], there is a chance that the extended LND did not capture all of the disease. A more recent study by Laganosky et al. in 2019 indicated statistically significant improved 5-year CSS and OS for patients with advanced RCC who underwent extended LND (removal of ≥10 lymph nodes) compared with those who had non-extended LND [26]. Better OS was also found for pT3N0M0 RCC patients who underwent more extensive LND, which Wei et al. designated as the removal of two or more lymph nodes [42]. Since the risk of LNM increases with increasing tumor stage [43,44], it is possible that increasing the extent of LND confers an oncologic benefit by reducing the overall tumor burden. While most of these studies suggest an association between LND extent and improved survival, the lack of a defined and standard template hinders comparability and applicability. For the studies that define the LND template used, it confirms that the primary landing sites for right-sided tumors are indeed the paracaval, precaval, retrocaval, and interaortocaval nodes, and for left-sided tumors, the paraaortic, preaortic, and retroaortic nodes.

Although there is some evidence suggesting a possible benefit of LND, the mechanism responsible for its therapeutic value is still unclear. These studies were based on observational data and were susceptible to selection bias, which is compounded by a lack of a standardized LND template and the subjectivity of the surgeon’s decision to perform the dissection. The limited evidence that supports LND are all retrospective studies, which limit control over study design and are prone to reverse causality. The small sample sizes in these studies also restrict the generalizability of these findings and may not be representative of the RCC patients at large. More importantly, a small sample size can negatively impact the sensitivity and comparability of these studies. The varying LND templates and lack of defined templates further impedes the ability to compare the study findings.

### 2.3. Evidence against Lymph Node Dissection and Related Complications

More recent investigations suggest that LND is not associated with improved outcomes and endorse the initial claims of the EORTC 30881 trial. A study in 2020 by Bacic et al. recapitulated the infamous trial and modeled a hypothetical trial comprised of patients at risk for nodal metastasis. It was suspected that LND bestows the greatest benefit for those at greatest risk for nodal disease, so emulating a clinical trial with a high-risk cohort was warranted. In their analyses, they confirmed the original findings of the EORTC 30881 trial and found no association between LND and improved OS [27]. In fact, a small, statistically significant increase in mortality risk was found in association with LND. This echoed the same results by Gershman et al., where they evaluated the relationship between LND and the development of distant metastases, CSM, and ACM with a propensity score-based analysis. They found that LND did not offer any oncologic benefit and, in contrast to previous reports, neither did its extent [28]. Marchioni et al. offered a similar conclusion and found that neither LND nor its extent protected patients from CSM [10]. Even among a high-risk cohort, described as those with radiographic lymphadenopathy or increased risk of nodal disease, LND was not associated with improved survival. Additionally, a systematic review of the literature in recent years revealed no benefit of LND, even for patients at increased risk of nodal disease, and may even reduce CSS [6,30]. A meta-analysis of the literature by Shi et al. also showed a declining trend in evidence supporting LND since 1979, which follows the decreasing rates of LND [20].

It was previously reported that the therapeutic benefit of LND could be due to an improved response to systemic therapy in the case of limited nodal disease [45]. Surely high-risk patients who receive adjuvant therapy in conjunction with LND will experience improved OS if this is true, but a secondary analysis of the Adjuvant Sorafenib and Sunitinib for Unfavorable Renal Carcinoma trial indicated otherwise, and no oncologic benefit was associated with LND [29]. Other studies maintain the same claim that LND does not hold any therapeutic value, regardless of the risk for nodal metastases. Patients with renal masses ≥7 cm showed no differences in recurrence-free survival (RFS) or OS, irrespective of receiving LND [8]. CSS, OS, and PFS also did not improve with LND for patients with pT3 disease [31]. Another study by Gershman et al. reproduced comparable results and found no association with improved oncologic outcomes among nonmetastatic RCC patients with radiographic lymphadenopathy or increasing risk of nodal disease [32]. A multi-institutional analysis evaluating the CSS, OS, and RFS also demonstrated no association with LND or its extent among patients at risk for nodal disease [33].

There is far more literature indicating no benefit of LND, especially within recent years, but an appropriate prospective trial is needed to determine whether or not it provides any advantage. The current literature is conflicting and offers varying conclusions, with very few studies reporting the indications for LND and its extent. The contradictory evidence can be attributed to a wide range of sample sizes and the overwhelming number of retrospective studies that limit the comparability among the studies. Future studies should specify the LND template used and describe its extent so that clinicians can better identify candidates for surgical resection and understand its role. However, the future of LND may become obsolete with the emergence of adjuvant therapies such as pembrolizumab, which has demonstrated significantly better outcomes for RCC patients with a high risk of recurrence [46]. As such, the rise in these new therapies is consistent with the declining rates of LND [20]. Even if does not confer a therapeutic benefit, LND is still an invaluable tool for risk stratification, which has improved patient selection for various postoperative adjuvant therapies [47]. Considering the risks of LND is equally as important as assessing its therapeutic benefit, especially when it is still debated. Some of the most common complications include major hemorrhage, bowel damage, and the development of chylous ascites [48,49]. The EORTC 30881 trial did not reveal any differences in complication rates among patients who underwent RN with LND and RN alone [4]. The surgical complications they analyzed in eligible patients included bleeding >1 L, pleural damage, infection, bowel damage, embolism, and lymph fluid drainage. Even though the rates of bleeding and embolism were higher in the former group compared with the latter, it was not statistically significant. Other studies also indicate that rates of perioperative complications do not differ significantly between the two groups [50,51]. These studies were not designed to compare complication rates, though, so they may have been underpowered to detect any statistically and clinically significant differences. However, Gershman et al. used a propensity score-based analysis to investigate the perioperative morbidity of LND at the time of nephrectomy and found that it was not associated with increased rates of major complications, which were defined as a Clavien grade 3 or above [52]. These studies all suggest that the complication rate is not significantly different between patients who receive RN with LND and those who undergo RN alone. However, it should be noted that LND templates and indications were heterogeneous. Furthermore, the observed complication rates also depended on the surgeon’s experience and the extent of the disease. The available evidence to date suggests that LND most likely does not increase the complication rate.

### 2.4. When to Perform Lymph Node Dissection

**Figure 2:**
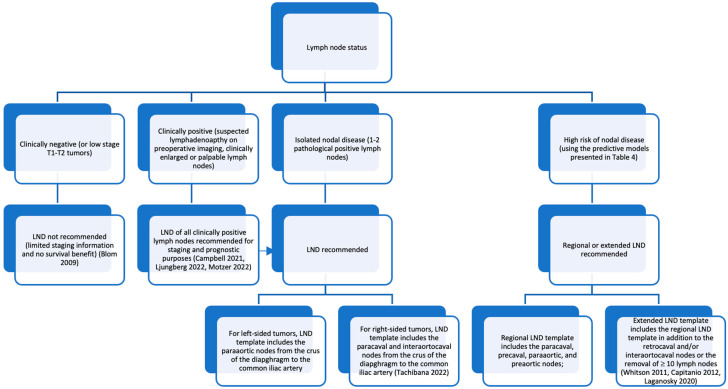
Patient selection for lymph node dissection (LND) at the time of nephrectomy and possible templates [4,11,12,13,22,23,24,26].

With the available contradicting evidence, it is important to delineate the role of LND in RCC and the selection of appropriate patients who will most likely receive the greatest benefit. Contemporary guidelines suggest performing LND only for RCC patients who present with clinically suspicious lymph nodes upon physical examination or radiographic imaging [11,12,13], but these recommendations do not offer any additional guidance on patient selection (Table 1). Since patients with RCC and LNI often portend a worse prognosis than those without LNI [7,53,54], it is reasonable to assume that those with or at risk for nodal disease will benefit the most from LND. Indeed, some studies have found that certain high-risk patients who receive LND have shown improved survival [21,22], but it is still unclear who those patients are. We created a recommendation for LND and possible templates for selecting patients based on current guidelines and supporting evidence as can be presented in Figure 2.

Current imaging techniques used for the early detection of LNM, including ultrasound, computed tomography (CT), and magnetic resonance imaging (MRI), have limited sensitivity and specificity. They have considerable rates of false negatives and false positives due to undetectable micrometastasis or reactive inflammation [5,48]. Newer imaging technologies, such as lymphotropic nanoparticle-enhanced MRI (LNMRI), are promising, but larger studies regarding their role in nodal staging are required. A comprehensive literature review by Tadayoni et al. evaluated multiple studies and the specificities and sensitivities of various imaging modalities: ultrasound, CT, multidetector-row CT (MDCT), MRI, LNMRI, and F-fluoro-2-deoxyglucose positron emission tomography (FDG-PET) (Table 3). Even though CT is relatively inexpensive and the most widely used, they recommend using MRI in tandem with FDG-PET because it may offer the highest accuracy [55]. The existing imaging technologies have limited application in detecting micrometastatic disease and identifying patients with normal-sized lymph nodes who may benefit from LND [56,57]. Improving the accuracy of current preoperative imaging and developing newer ones will enhance nodal staging and outline the role of LND in RCC.

If the surgical resection of lymph nodes truly confers an advantage, a more reliable method of identifying these high-risk patients should be prioritized. Several predictive models have been developed to accomplish this task and are summarized in Table 4. In 2004, Blute et al. used the primary pathological features of patients with nonmetastatic sporadic clear cell RCC to predict the probability of nodal disease and noted several intraoperative risk factors. In their assessment, an increased risk of LNM was found in patients who had tumors described as nuclear grade 3–4, with sarcomatoid components, size ≥ 10 cm, stage pT3 or pT4, or coagulative tumor necrosis [58]. Patients with an increasing number of these features demonstrated a significantly higher risk of nodal disease, where the risk increased from 0.4% with no features to 4.4% with two features and over 50% with all five features. These are considerable findings but are limited in application because these features can only be assessed perioperatively. Hutterer et al. developed a nomogram with 78.4% accuracy that included patient age, radiographic tumor size, and symptom classification [59]. These symptoms were previously described by Patard et al. and classified as asymptomatic, local, or systemic [60]. Local symptoms included lumbar pain, hematuria, or palpable mass, and systemic symptoms included anorexia, asthenia, weight loss, or symptoms due to metastasis. The presence of systemic symptoms was associated with a greater risk of LNM compared with the presence of local symptoms. However, this nomogram was based on hilar node sampling, which is not a primary landing site for RCC and may underestimate the actual risk of LNM. Their nomogram could also be refined to include more risk factors specifically related to nodal disease. Capitanio et al. developed another model with 86.9% accuracy using exclusively clinical factors [61]. Their data showed tumor stage, clinical nodal status, metastases at diagnosis, and clinical tumor size to be the most important predictors of LNM. Another predictive model by Babaian et al. used the presence of lumbar pain or hematuria, ECOG performance status, clinical nodal stage, and lactate dehydrogenase to estimate the probability of LNM and showed 89% discrimination [62]. One study by Gershman et al. suggested that preoperative radiographic imaging is also an important independent predictor of nodal disease, where maximum lymph node short axis diameter and perinephric/sinus fat invasion showed high predictive value [63]. The most recent nomogram from Li et al. incorporates physical examination, lab parameters, and preoperative imaging and has reliable predictive value. Their nomogram includes the age at surgery, clinical tumor stage, clinical nodal stage, lymphocyte percentage, and presence of lumbar pain, hematuria, and a palpable mass [64].

### 2.5. Sentinel Lymph Node Biopsy

Sentinel lymph node biopsy (SLNB) is an encouraging alternative to LND for nodal staging. It has proven valuable in other cancers, such as breast and penile cancer [65,66]. However, its role in RCC is unclear due to the unpredictability of renal lymphatics. Generally, SNLB involves the injection of a radioactive tracer near or at the primary tumor, followed by detection with a gamma probe. At present, only a few studies have evaluated its utility in the detection of LNM, but it has demonstrated long-term safety and low morbidity in patients with RCC [67]. The detection rate of LNM with SNLB ranges between 61–82% [68,69,70], but these results are restricted by small sample sizes. SNLB has potential for improvement with modifications, such as PET/CT or near infra-red fluorescence optical imaging agents [71], but its therapeutic implication in high-risk patients is still unknown and requires standardization. Its use will remain experimental until larger prospective studies are conducted.

## 3. Future Direction

With no clear, proven benefit, the future of LND in the surgical management of nonmetastatic localized RCC is uncertain. Deciphering the complexities of renal lymphatic drainage will be key to locating sites of LNM and outlining accurate templates. This will improve disease staging and existing nomograms for identifying patients at risk for nodal metastasis, who are believed to benefit the most from LND. In addition to identifying these patients, future randomized studies could use the proposed nomograms for establishing high-risk cohorts to gain better insight into the therapeutic value of LND. They should also define the extent of LND, especially since most findings from retrospective studies have been based on undefined templates. Future studies should also have larger sample sizes to improve generalizability and sensitivity and reduce bias.

Using potential biomarkers to predict LNM might also lead to the development of more accurate predictive models. A retrospective study by Kroeger et al. found that low levels of carbonic anhydrase IX and high epithelial vascular endothelial growth factor 2 protein expression were associated with a higher risk of LNM [72]. Identifying additional genetic, tissue, or urine biomarkers has proven to be challenging due to the significant intra-tumoral heterogeneity of renal masses. However, the growing use of artificial intelligence (AI) in the prediction of LNM bodes a promising future for LND. It has not only shown high predictive accuracy of LNM in patients with RCC [73], but has also offered accurate detection of positive lymph nodes and prognostic information in patients with prostate cancer based on PET/CT interpretation [74]. The role of machine learning is also growing in pathology for various cancers, such as breast and skin cancers, serving as an important prognostic and diagnostic tool [75]. Advancements in AI will likely be applicable to the detection of nodal disease in RCC and perhaps redefine the role of LND, offering preoperative diagnosis and intraoperative guidance. Furthermore, the utility of LND in the various histological subtypes of RCC is still unknown. The rate of LNM varies with histology and should be considered when selecting patients for LND. According to one study, patients with chromophobe RCC had the lowest rate of LNI (7%), and those with collecting duct RCC had the highest (71.4%) [76]. Since histology has major implications on LNM and prognosis, more studies should investigate the relationship between LND and different RCC subtypes. Nomograms that assess the probability of nodal disease in nonmetastatic RCC patients should also consider including histology, but the increasing number of subtypes being discovered poses a significant challenge. Future studies should also explore the contributions of LND in sporadic forms compared with hereditary forms of RCC because robust data are lacking, and each form is associated with different risk factors. To date, there are no recommendations for LND in patients with different forms of sporadic RCC. Studies evaluating the oncologic role of LND may benefit from further RCC stratification by creating cohorts with sporadic or hereditary RCC and/or different histological subtypes.

## 4. Summary and Conclusions

Despite the clear staging and prognostic role of LND in the management of RCC, its therapeutic value remains uncertain or conflicting at best. Initial evidence supporting LND has been clouded by retrospective studies with inconsistent indications and templates, which continue to impede current studies. The lack of a standard LND template makes it challenging to interpret and compare the findings in various studies. The only prospective, randomized trial, EORTC 30881, showed no oncologic advantage for patients who underwent LND with nephrectomy compared with those who did not undergo LND [4]. While an association was found between LND and improved survival in some of the studies, they were influenced by selection bias and relied on retrospective analyses. There is an inherent selection bias in the investigation of LND since the patients selected for LND already have a worse prognosis due to nodal disease. The studies were also limited by particularly small sample sizes, which reduce the statistical power and external validity of these studies. LND may confer an advantage in a select group of patients, but they have yet to be identified, and the evidence is limited and dated. Most contemporary studies have not found any associated survival benefit with LND and overshadow the handful of studies that do report an oncologic advantage. The variability in sample size and study type among the studies also makes it more difficult to compare the results. Even if LND does not end up showing an oncologic benefit, it remains an indispensable procedure in pathologic nodal staging and prognostication. It may perhaps improve the selection of high-risk patients for systemic adjuvant therapy.

The conflicting evidence for LND in the setting of nonmetastatic localized RCC calls for larger, prospective studies to improve statistical power and external validity of studies that evaluate the benefit of LND. The incorporation of machine learning in the detection of nodal disease and prediction of LNM will offer better prognostic information for RCC patients and may shape the future of LND. It can also guide LND templates and result in better sampling of positive nodes, perhaps dispelling the complexities of renal lymphatics and controversy surrounding the benefit of LND.

The studies evaluating the therapeutic role of LND have also been plagued by a lack of clear guidelines regarding when and how it needs to be performed and the unpredictable nature of renal lymphatics. Revising the current cancer staging system to group patients with node-positive RCC and those with metastatic RCC together may improve risk stratification and allow clinicians to select high-risk patients for further treatment. For now, with emerging new therapies and alternatives to LND, it is no surprise that a significant decrease in the rates of LND has been observed and the decline will probably continue until more robust data are available.

## Figures and Tables

**Table 1:** Summary of the current guidelines involving lymph node dissection (LND) from the American Urological Association (AUA), the National Comprehensive Cancer Network (NCCN), and the European Association of Urology (EAU).

Reference	Year	Recommendations for LND
AUA [11]	2021	LND should include all clinically positive lymph nodes for patients undergoing surgical excision of renal masses with clinically suspicious lymphadenopathy
NCCN [12]	2022	LND at the time of surgery for patients with palpable or visibly enlarged lymph nodes or lymphadenopathy on preoperative imaging
EAU [13]	2022	LND is not recommended for patients with organ-confined disease; LND during nephrectomy should remove clinically enlarged lymph nodes for staging, prognosis, and follow-up implications

**Table 2:** Studies evaluating the therapeutic value of lymph node dissection (LND) in the management of renal cell carcinoma (RCC) and their main findings.

Reference	Country	Journal	Year Published	Study Type	Study Duration	Sample Size	Study Population	Intervention	Main Findings
Gershman [21]	USA	*European Urology*	2017	Retrospective	1980–2010	138	pN1M0	PN or RN with LND	Patients who experienced MFS 5 years after LND also experienced MFS at longer follow-up: 5- and 10-year MFS (16 and 10%), CSS (26 and 21%), and OS (25 and 15%)
Tachibana [22]	USA	*Urologic Oncology*	2022	Retrospective	2000–2019	45	pTanyN0-1M0	Nephrectomy with limited * or template LND	Template LND (left-side paraaortic nodes from crus of diaphragm to common iliac artery and right-side paracaval and interaortocaval nodes with the same boundaries) associated with improved 5-year OS in patients with disease limited to 1–2 LNs (60.3% compared with 39.3% for patients who underwent limited LND)
Whitson [23]	USA	*Journal of Urology*	2011	Retrospective	1988–2006	9586	pTanyNanyM0	PN or RN with LND	LND extent associated with improved CSS for patients with pN1 disease: 5-year predicted probability of DSS is 39% in patients with 5 LNs removed and 49% in patients with 15 LNs removed; 10% absolute increase in DSS at 5 years when LND is increased to 10 nodes in a patient with 1 positive node
Capitanio [24]	Italy	*Urologia Journal*	2012	Retrospective	1987–2011	44	pT4	Nephrectomy with regional (hilar region and right-side precaval or left-side paraaortic nodes from adrenal vein to aortic bifurcation and interaortocaval nodes) or extended LND	Extended LND (left-side paraaortic and preaortic nodes from crus of diaphragm to aortic bifurcation and right-side retro/precaval nodes from adrenal vein to aortic bifurcation and interaortocaval nodes) associated with decreased CSM (HR 0.84) Removal of each additional LN resulted in 8% decrease in risk of dying
Capitanio [25]	Italy	*BJU International*	2014	Retrospective	1987–2011	1983	pTanyNanyMany	PN or RN with no LND, limited LND (ipsilateral hilar nodes), regional LND (limited LND plus right side pre-retrocaval nodes or left side paraaortic nodes), or extended LND (regional LND plus interaortocaval nodes)	LND extent associated with improved CSS (3–18% increase) in specific subgroups with: pT2a-pT2b tumors (HR 0.91) pT3c-pT4 tumors (HR 0.89) >10 cm tumors (HR 0.97) Sarcomatoid component (HR 0.81) Removal of each additional LN resulted in a 3–11% decrease in risk of metastatic progression
Laganosky [26]	USA	*Asian Journal of Urology*	2020	Retrospective	2004–2015	4397	T3-4NanyM0	PN or RN with extended LND or non-extended LND	Extended LND (≥10 LNs removed) associated with improved 5-year CSS (61.4% compared with 55.2% for those with non-extended LND) and OS (59.2% compared with 51.1% for those with non-extended LND) in patients with T3b-T3c disease Extended LND associated with improved 5-year OS (50.0% compared with 30.1% for those with non-extended LND) in patients with T4 disease
Bacic [27]	USA	*Urology*	2020	Retrospective	2004–2013	EORTC 30881 Trial Emulation 67388 High-risk Trial Emulation 69477	cT1-3cN0cM0 cT1-4cN0-1cM0	RN with or without LND	LND not associated with improved OS in both trial emulations
Gershman [28]	USA	*European Urology*	2017	Retrospective	1990–2010	1797	pTanyNanyM0	RN with or without LND	LND not associated with decreased risk of distant metastases, CSM, or ACM
Ristau [29]	USA	*Journal of Urology*	2018	Prospective	2006–2010	1943	pTanyNanyM0	PN or RN with or without LND	LND not associated with improved OS; LND associated with worse DFS
Marchioni [10]	USA	*BJU International*	2018	Retrospective	2001–2013	25357	pT2-3NanyM0	RN with or without LND	LND extent associated with small decrease in CSM in patients with positive nodes
Shi [30]	Multiple	*Frontiers in Oncology*	2021	Systematic review and meta-analysis	Variable	135514	TanyNanyM0	Nephrectomy with or without LND	LND not associated with improved OS; LND associated with a negative effect on CSS
Feuerstein [8]	USA	*World Journal of Urology*	2014	Retrospective	1990–2012	524	pT2-4N0-1M0	PN or RN with or without LND	LND not associated with improved DFS or OS
Li [31]	China USA	*Journal of Cancer*	2019	Retrospective	2006–2013 2010–2014	245 182	pT3	RN with or without LND	LND not associated with improved PFS, CSS, or OS; extended LND associated with worse OS
Gershman [32]	USA	*Journal of Urology*	2018	Retrospective	1990–2010	2722	pTanyM0	RN with or without LND	LND not associated with decreased CSM or ACM but associated with increased risk of distant metastases in overall cohort; LND not associated with decreased risk of distant metastases, CSM, or ACM in patients with cN1 disease or increasing probability thresholds of pN1 disease
Kokorovic [33]	Canada	*Urologic Oncology*	2021	Retrospective	2011–2019	2699	TanycN0-1M0	RN with or without LND	LND not associated with improved OS, RFS, or CSS, even in patients with increasing probability thresholds of pN1 disease; LND associated with increased risk for mortality; LND extent not associated with improved OS or CFS; LND extent associated with worse RFS
Blom [4]	Europe	*European Urology*	2009	RCT	1988–1991	772	T1-3	RN with or without LND	LND not associated with improved OS or PFS

CSS: cancer-specific survival; DSS: disease-specific survival; HR: hazard ratio; LN: lymph node; MFS: metastasis-free survival; OS: overall survival; RN: radical nephrectomy; PN: partial nephrectomy; RCT: randomized controlled trial; and RN: radical nephrectomy. * not defined.

**Table 3:** The sensitivities and specificities of the various imaging modalities evaluated by Tadayoni et al. [53] when evaluating detection of lymph node metastasis.

Modality	Sensitivity (%)	Specificity (%)
Ultrasound	100	Not reported
CT	60–100	75–98.1
MDCT	75–77	75–82
MRI	100	92
LNMRI	100	95.7
FDG-CT	75–87	100

**Table 4:** The parameters and accuracy of the various predictive models used to identify patients at risk for nodal disease.

Model	Year Published	Parameters	Accuracy	Risk of Lymph Node Metastasis
Blute [58]	2004	Number of features: Nuclear grade 3 or 4 Presence of sarcomatoid component Tumor size ≥ 10 cm Tumor stage pT3 or pT4 Presence of histological tumor necrosis	Not reported	Number of features present—risk of LNM: 0–0.4% 1–1.0% 2–4.4% 3–12.4% 4–13.2% 5–53.3%
Hutterer [59]	2007	Nomogram using: Age Tumor size Symptom classification (local vs. asymptomatic and systemic vs. asymptomatic)	78.4%	Presence of local symptoms showed 2-fold increase in LNM rate and presence of systemic symptoms showed 2.8-fold increase in LNM rate
Capitanio [61]	2013	Nomogram using: Clinical T3–T4 Clinical nodal status Metastasis at diagnosis Clinical tumor size	86.9%	T3-4 vs. T1-2 showed a 1.5-fold increase in LNM rate Clinical nodal status (cN1 vs. cN0) showed a 7-fold increase in LNM Tumor size shown to have a significant association as well
Babaian [62]	2015	Nomogram using: Local symptoms ECOG performance status Clinical nodal stage Lactate dehydrogenase	89%	
Gershman [63]	2016	Clinical and radiographic features: Maximum lymph node short-axis diameter Radiographic perinephric/sinus fat invasion	85%	Maximum lymph node short axis diameter (OR 1.19) Radiographic perinephric/sinus fat invasion (OR 44.64)
Li [64]	2019	Nomogram using: Age at surgery Clinical tumor stage Clinical nodal stage Lymphocyte percentage Clinical symptoms (lumbar pain, hematuria, or palpable mass)	82.4%	

LNM: lymph node metastases; OR: odds ratio.

## Data Availability

Not applicable.
